# Synergism of Proneurogenic miRNAs Provides a More Effective Strategy to Target Glioma Stem Cells

**DOI:** 10.3390/cancers13020289

**Published:** 2021-01-14

**Authors:** Adam Kosti, Rodrigo Barreiro, Gabriela D. A. Guardia, Shiva Ostadrahimi, Erzsebet Kokovay, Alexander Pertsemlidis, Pedro A. F. Galante, Luiz O. F. Penalva

**Affiliations:** 1 Greehey Children’s Cancer Research Institute, University of Texas Health Science Center at San Antonio, San Antonio, TX 78229, USA; kosti@uthscsa.edu (A.K.); rbarreiro@mochsl.org.br (R.B.); ostadrahimi@uthscsa.edu (S.O.); pertsemlidis@uthscsa.edu (A.P.); 2 Department of Cell Systems & Anatomy, University of Texas Health Science Center at San Antonio, San Antonio, TX 78229, USA; kokovaye@uthscsa.edu; 3 Centro de Oncologia Molecular, Hospital Sirio-Libanes, São Paulo 01308-060, Brazil; gguardia@mochsl.org.br (G.D.A.G.); pgalante@mochsl.org.br (P.A.F.G.); 4 Departamento de Bioquimica, Instituto de Quimica—Universidade de São Paulo, São Paulo 05508-000, Brazil; 5 Department of Pediatrics, University of Texas Health Science Center at San Antonio, San Antonio, TX 78229, USA

**Keywords:** miRNA, glioblastoma, neuroblastoma, miR-124, miR-128, miR-137

## Abstract

**Simple Summary:**

miRNAs function as critical regulators of gene expression and have been defined as contributors of cancer phenotypes by acting as oncogenes or tumor suppressors. Based on these findings, miRNA-based therapies have been explored in the treatment of many different malignancies. The use of single miRNAs has faced some challenges and showed limited success. miRNAs cooperate to regulate distinct biological processes and pathways and, therefore, combination of related miRNAs could amplify the repression of oncogenic factors and the effect on cancer relevant pathways. We established that the combination of tumor suppressor miRNAs miR-124, miR-128, and miR-137 is much more effective than single miRNAs in disrupting proliferation and survival of glioma stem cells and neuroblastoma lines and promoting differentiation and response to radiation. Subsequent genomic analyses showed that other combinations of tumor suppressor miRNAs could be equally effective, and its use could provide new routes to target in special cancer-initiating cell populations.

**Abstract:**

Tumor suppressor microRNAs (miRNAs) have been explored as agents to target cancer stem cells. Most strategies use a single miRNA mimic and present many disadvantages, such as the amount of reagent required and the diluted effect on target genes. miRNAs work in a cooperative fashion to regulate distinct biological processes and pathways. Therefore, we propose that miRNA combinations could provide more efficient ways to target cancer stem cells. We have previously shown that miR-124, miR-128, and miR-137 function synergistically to regulate neurogenesis. We used a combination of these three miRNAs to treat glioma stem cells and showed that this treatment was much more effective than single miRNAs in disrupting cell proliferation and survival and promoting differentiation and response to radiation. Transcriptomic analyses indicated that transcription regulation, angiogenesis, metabolism, and neuronal differentiation are among the main biological processes affected by transfection of this miRNA combination. In conclusion, we demonstrated the value of using combinations of neurogenic miRNAs to disrupt cancer phenotypes and glioma stem cell growth. The synergistic effect of these three miRNA amplified the repression of oncogenic factors and the effect on cancer relevant pathways. Future therapeutic approaches would benefit from utilizing miRNA combinations, especially when targeting cancer-initiating cell populations.

## 1. Introduction

MicroRNAs (miRNAs) are small noncoding RNAs that act as essential post-transcriptional regulators [1] and are implicated in functions ranging from development to homeostasis [2]. miRNAs are especially important in cancers, where many have been identified as oncogenes or tumor suppressors [3]. Dysregulation of tumor-suppressive miRNAs is associated with the aggressive and undifferentiated nature of neural-derived cancers [4,5]. Glioblastoma multiforme (GBM) and neuroblastoma arise from transformed neural precursors [6,7], where normal stem cell programs are taken over. Therefore, the use of proneurogenic miRNAs as agents to induce terminal differentiation and turn off oncogenic pathways has been proposed as an option to treat these tumors [8]. In the brain, miRNAs control processes such as neuronal differentiation, neuronal processes, and regional specialization [9]. Marked changes in miRNA profiling have been observed during neurogenesis and in comparisons between undifferentiated and differentiated cells [10]. Among brain enriched miRNAs, miR-124-3p, miR-128-3p, and miR-137-3p (hereinafter miR-124, miR-128, and miR-137) stand out. Decreased expression of these three tumor suppressor miRs occur in different cancers [11,12]. miR-124 is known for its high abundance, accounting for most miRNAs in a neuron [8] and plays roles in CNS development, neurodegeneration, CNS stress, stroke, and neuroimmunity [13]. miR-128 has been connected to Huntington’s disease [14] and associated with anxiety disorders [15]. miR-128 increases expression during brain development, leading to repression of nonsense mediated decay machinery and upregulation of mRNAs normally targeted for decay. miR-137 is a well-characterized marker of schizophrenia susceptibility [16] and is downregulated in brain tissue from patients with depression and suicidal behavior [17].

We have previously shown that miR-124, miR-128, and miR-137 have similar patterns of expression during neurogenesis. When combined, these three miRNAs have a much stronger effect on differentiation and proliferation, suggesting that they act synergistically to coordinate adult neuronal differentiation [18]. This synergistic effect can be explained based on their target sets, which are highly overlapping and also connected via networks of associated genes, with transcription factors forming important regulatory interactions [18]. In cancer, downregulation of these three miRNAs contributes to the acquisition of a less differentiated phenotype. Multiple studies in different tumor types have shown that transfection of mimics of these three miRNAs affects several cancer-relevant phenotypes and block tumor growth [11,19,20].

Most preclinical and clinical studies using miRNA mimics or antagomiRs as therapeutic agents are designed around a single miRNA [21]. This approach poses many limitations, including the amount of agent required to have a therapeutic effect and reduced regulatory impact on target genes. As in the example of miR-124, miR-128, and miR-137, many other miRNAs work cooperatively or synergistically to regulate critical biological processes [22]. Taking advantage of these regulatory interactions, there is growing interest in the concept of using miRNA combinations in cancer therapy [23]. In this study, we show that the combination of miR-124, miR-128, and miR-137 works more effectively than single miRNA. Genomic analyses established that this miRNA combination downregulated target genes implicated preferentially in transcription regulation, metabolism, neuronal differentiation, and angiogenesis. Finally, we show that miR-124, miR-128, and miR-137 belong to a larger network of proneurogenic tumor suppressor miRNAs; thus, other miRNA combinations also could be effective in targeting cancer cells, expanding treatment options.

## 2. Results

### 2.1. Establishing miRNA Synergism as a Tool for Therapy

**Figure 1:**
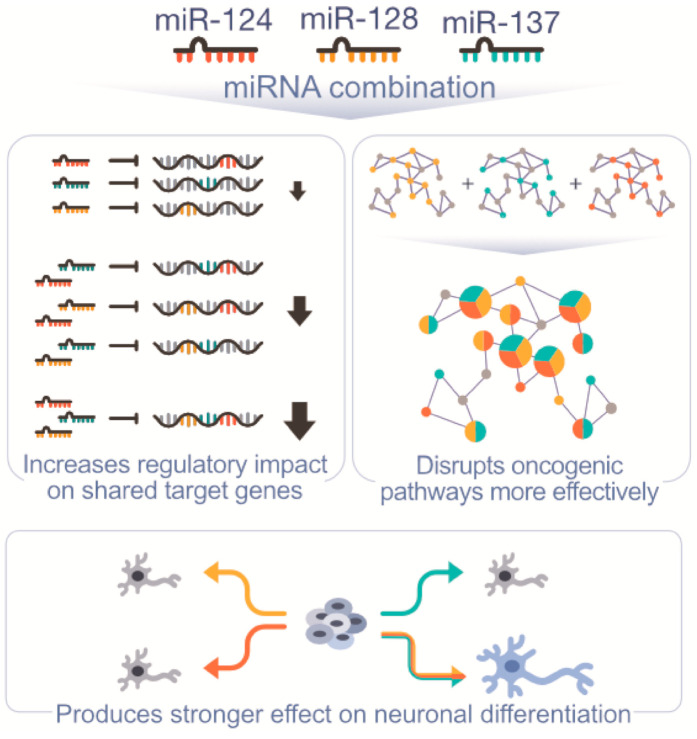
miR-124, miR-128, and miR-137 synergism. Model for synergistic interactions between miR-124, miR-128, and miR-137. Combined action of the three miRNAs on shared and associated targets produces a stronger regulatory effect. This includes a stronger repression of shared targets, increased effect on neuronal differentiation, and broader and stronger impact on oncogenic pathways.

**Figure 2:**
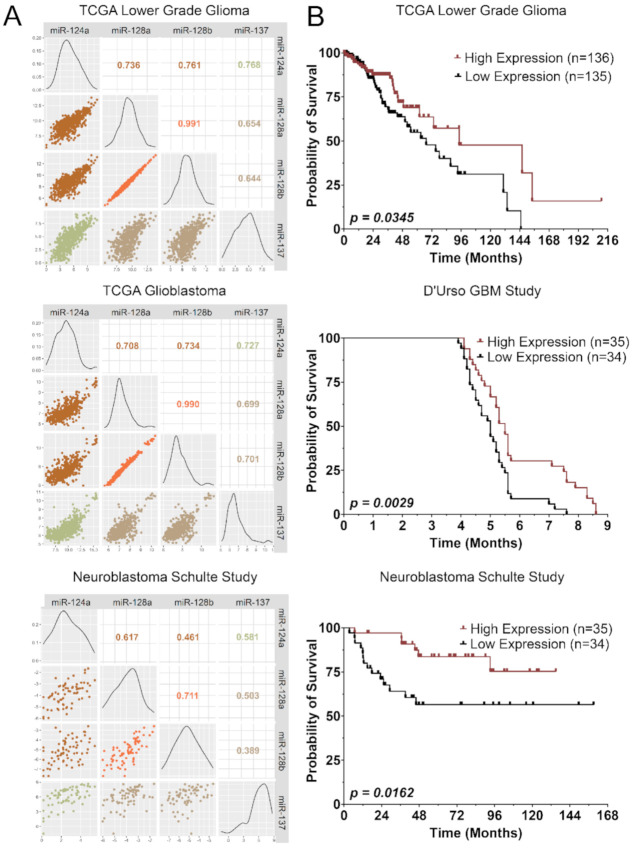
miR-124, miR-128, and miR137 coexpression in glioma and neuroblastoma patients and impact on patient survival. (**A**) miRNA expression correlation in patient samples from the The Cancer Genome Atlas Low Grade Glioma (TCGA LGG), The Cancer Genome Atlas Glioblastoma Multiforme (TCGA GBM), and in neuroblastoma (Schulte study). Upper right panels display Pearson correlation coefficient between the miRNAs. Middle panels display distribution of miRNA expression. Lower left panels display miRNA expression scatterplots. (**B**) Survival rates of patients expressing low vs. high miR-124, -128, and -137 in TCGA LGG, D’Urso GBM Study, and Schulte Neuroblastoma Study.

We have previously shown that miR-124, miR-128, and miR-137 share a large set of target genes and work synergistically to promote neuronal differentiation (Figure 1, Table S1) [18]. Moreover, their expression patterns during neurogenesis and in healthy brain tissue suggest that they are coexpressed and coregulated [18]. Similarly, analyses of TCGA LGG, GBM, and neuroblastoma miRNA profiling studies showed that these three miRNAs display strong correlations of expression (Figure 2A) [24]. Agreeing with their role as tumor suppressors, higher overall expression of these three miRNAs based on z-score average was associated with better prognosis (Figure 2B) [25].

**Figure 3:**
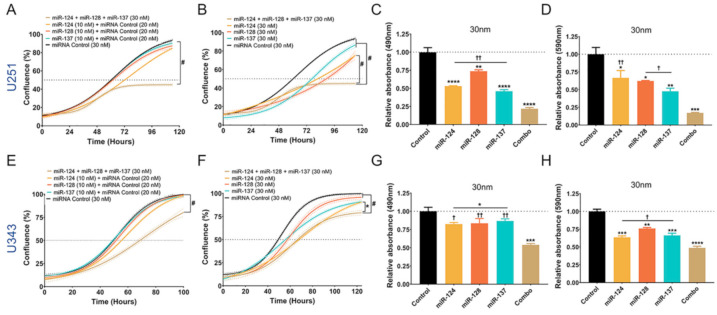
Synergistic effects of miR-124, miR-128, and miR-137 on glioblastoma cells. (**A**,**E**) Cell proliferation with live-cell imaging (Incucyte) of U251 and U343 cells following reverse transfection with low concentrations (10 nM) of individual miRNAs and combination of the three miRNAs (total 30 nM). Effects of the combination were greater than expected additivity, indicating synergy based on linear and Bliss models (U251 Linear CI: 0.339; Bliss CI: 0.338; U343 Linear CI: 0.152; Bliss CI: 0.151). Tukey test for significance at 120 h, # = *p* < 0.0001. (**B**,**F**) Effects of miRNA combination on cell proliferation versus individual miRNAs at an equivalent concentration (30 nM). Tukey test for significance at 120 h, # = *p* < 0.0001. (**C**,**G**) Viability of GBM cells 48 h after reverse transfection with individual miRNA mimics and the combination (30 nM). (**D**,**H**) Clonogenic ability of GBM cells after reverse transfection with individual miRNA mimics and the combination (30 nM). A one-way ANOVA with Tukey test for multiple comparisons was utilized for experiments (**C**,**D**,**G**,**H**). p-values for comparisons against miRNA control: * *p* < 0.05; ** *p* < 0.01; *** *p* < 0.001; **** *p* < 0.0001. p-values for comparisons against miRNA Combo: † *p* < 0.05; †† *p* < 0.01.

miR-124, -128, and -137 individually display antitumorigenic activity [11,12]. Capitalizing on our previous findings, we examined whether the combination of the three miRNAs was more effective in targeting cancer cells than single miRNAs. Based on observed associations between these three miRNAs, we expected they would have a stronger impact on shared targets and robust repression of critical pathways based on the functional relationship of their target sets (Figure 1). We first tested their functional synergy in GBM cell lines using the response additivity model (or linear interaction effect) [26]. Low doses of each miRNA individually produced no significant changes in proliferation; however, when the three low doses were combined, they significantly inhibited proliferation based on live-cell imaging, indicating synergy with a combination index (CI) of 0.339 (Figure 3A,E). Using a more common model, the Bliss independence model [26], the selected miRNAs also strongly synergized, with CI of 0.338 for U251 and 0.151 for U343 at 120 h. Next, we transfected GBM cells with the same equimolar amount of miRNA mimics (single miRNA and miRNA combination) and tested whether effects of the miRNA combination vs. single miRNA mimics on GBM cells supported our model of synergism (Figure S1A). In both GBM lines, the miRNA combination decreased proliferation more significantly than any single miRNA at the same dose (Figure 3B,F). In addition, the combination inhibited glioma cell viability (Figure 3C,G) and clonogenic potential (Figure 3D,H) more significantly than any miRNA by itself.

**Figure 4:**
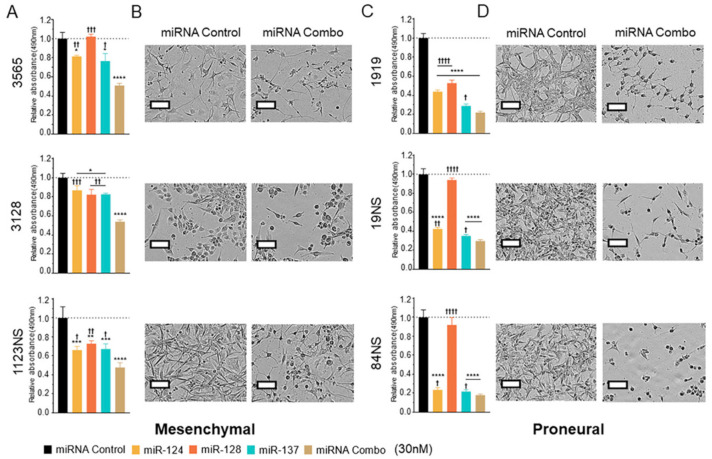
miRNA combination inhibits glioma stem cell phenotype. (**A**) Mesenchymal glioma stem cell (GSC) viability 120 h after reverse transfection with 30 nM of single and combined miRNA mimics. (**B**) Phenotypic changes of GSCs in (**A**). (**C**) Proneural GSC viability 120 h after reverse transfection with 30 nM of single and combined miRNA mimics. (**D**) Phenotypic changes of GSCs in (**C**). A one-way ANOVA with Tukey test for multiple comparisons was utilized for all experiments. *p*-values for comparisons against miRNA control: * *p* < 0.05; ** *p* < 0.01; *** *p* < 0.001; **** *p* < 0.0001. *p*-values for comparisons against miRNA Combo: † *p* < 0.05; †† *p* < 0.01; ††† *p* < 0.001; †††† *p* < 0.0001. Scale bar represents 100 µm.

Recurrence is the most frequent cause of GBM mortality [27]. Tumor-initiating cells or GSCs evade initial treatments and result in recurrence, making them an important cell population to target [28]. Previously, the three miRNAs had been explored individually as tumor suppressors in GSC cultures [11,12]; however, it was unknown whether they would synergize as seen in the GBM cell lines (Figure 3). We tested the efficacy of the miRNA combination in six GSCs with two distinct molecular subtypes (mesenchymal and proneural). Using the same total molecular amount, we observed in all six lines that miRNA combination had a stronger impact on cell viability than individual miRNAs and induced morphologic changes (Figure 4).

The miRNA combination also displayed significant effects on the proliferation of neuroblastoma BE(2)C and Kelly cells (Figure S1A). The combination demonstrated synergy based on the response additivity model CI of 0.355 and 0.264 for each cell line, respectively (Figure S2A). A hallmark of neuroblastoma cells is their ability to differentiate into benign neuron-like cells with long neurites and small cell bodies [29]. Differentiation agents like retinoic acid are often used to treat high-risk neuroblastoma [30]. We used a morphologic assay to assess differentiation [31], and found that the miRNA combination was more effective than individual miRNAs in driving neuroblastoma cell differentiation, based on increases in neurite length (Figure S2B,C).

### 2.2. Combined Impact of miR-124, -128, and -137 on Gene Expression

**Figure 5:**
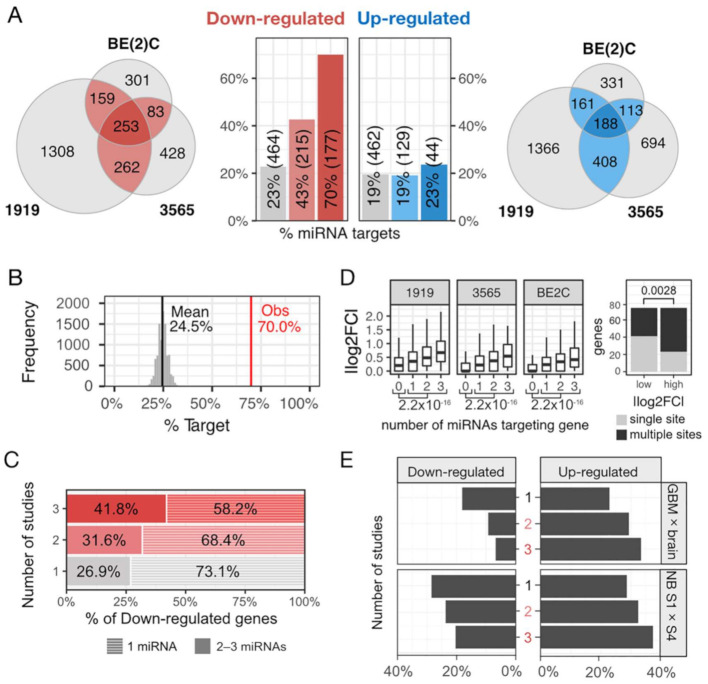
RNA-seq analysis of cells transfected with control and miRNA combo mimics. (**A**) Overlap between results of RNA-seq studies done in BE(2)C, GSCs 1919, and 3565 transfected with control vs. miRNA combination (miR-124, -128, and -137) mimics. Bar graphs: percentage of genes targeted by at least one of the transfected miRNAs identified in one, two, or all studies. Considering Venn-diagrams of down- and upregulated genes. As expected, we found an over-representation (253) in the downregulated genes (*p*-value = 0.000000602; 1.24-fold enrichment; Hypergeometric test) and an under-representation (188) of upregulated genes (*p*-value = 0.0000006; Hypergeometric test) shared by the three cell lines. (**B**) Number of genes targeted by at least one of the three transfected miRNAs appearing in the overlap of the three studies is much higher than expected by chance (*p*-value = 6.83 × 10^−66^). (**C**) Percentage of genes targeted by two or three of the transfected miRNAs appearing in the overlap of the three studies is much higher than the number observed in single studies. (**D**) Left: Genes targeted by all three miRNAs show greater decrease in expression versus genes targeted by two or a single miRNA. Right: Genes with multiple miRNA binding sites displayed higher silencing level in comparison to genes with a single miRNA binding site. (**E**) Percentage of miRNA target genes in all three, two, or single studies with increased expression in GBM in relation to normal brain (cortex) and stage 4 neuroblastoma compared to stage 1.

To understand the effects of combined miR-124, miR-128, and miR-137 mimics on gene expression, we performed RNA-seq in mesenchymal and proneural GSCs and neuroblastoma BE(2)C cells (Figure S1B). Detailed results are provided in Tables S2 and S3. In the downregulated sets, 757 genes appeared in at least two out of the three studies (Figure 5A). Based on our miRNA target list (Table S1), we determined that the percentage of genes containing binding sites for at least one of the three selected miRNAs was much higher in the overlap set compared to the gene set downregulated in a single cell line (Figure 5A). Of the 757 genes, 392 contained binding sites for at least one of the transfected miRNAs. The proportion of targets of miR-124/128/137 in the overlap was also higher than in the upregulated set and what would be expected by chance according to a permutation analysis (Figure 5A,B). We also observed more genes containing binding sites for two or three of the transfected miRNAs in the overlap compared to the gene set downregulated in a single cell line (Figure 5C). Supporting our definition of synergism, we observed that genes targeted by all three miRNAs tended to have greater changes in expression than genes targeted by two or a single miRNA. Results were similar in all three individual analyses (Figure 5D).

We next explored the biological nature of the 392 miRNA targets present in the overlap of our analyses. Since miR-124, miR-128, and miR-137 are tumor suppressor miRNAs and tend to be downregulated in tumors, we expected that their targets would show the opposite behavior. In GBM and neuroblastoma datasets, the miRNA targets present in all three analyses showed increased expression in GBM than in normal brain and higher expression in stage IV than in stage I neuroblastoma compared to miRNA targets present in two or in a single analysis (Figure 5E, Table S4).

**Figure 6:**
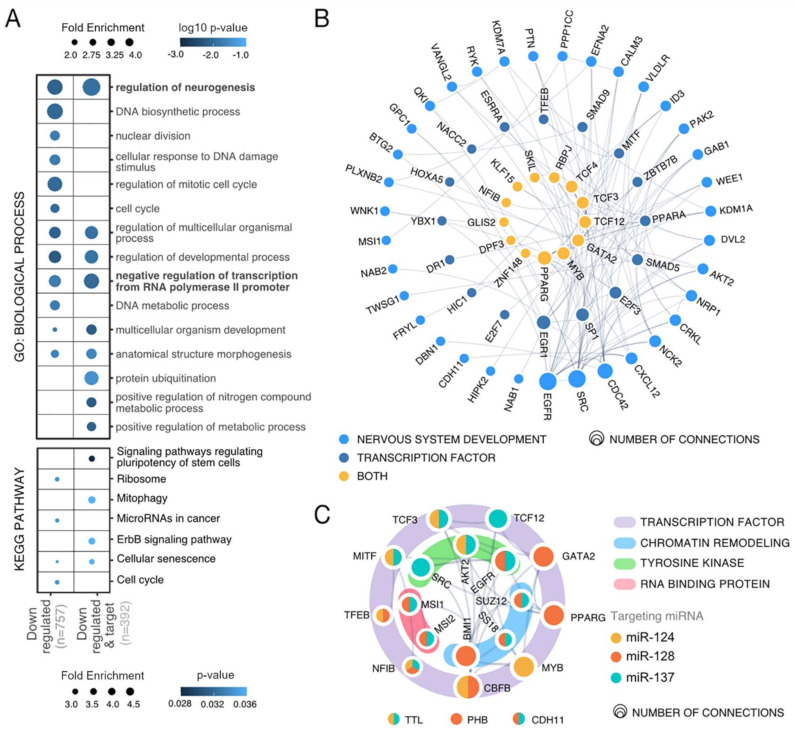
Gene ontology analysis of downregulated genes upon miRNA combo transfection. (**A**) Enriched biological processes and KEGG pathways identified by PANTHER and ShinyGO [32,33] associated with downregulated genes observed in at least two RNA-Seq analyses. (**B**) Network showing detected targets of the transfected miRNAs appearing in at least two RNA-Seq studies implicated in nervous system development and transcription regulation according to STRING [34]. (**C**) Oncogenes identified as targets of miR-124, miR-128, and miR-137 in the genomic analysis and their functions.

We examined biological processes and pathways preferentially associated with the miRNA target genes present in the overlap. Among the GO categories that appeared more often were those associated with development, neuronal differentiation, transcription regulation, and metabolism (Figure 6A, Tables S4 and S5). In a previous analysis to evaluate the individual impact of miR-124, miR-128, and miR-137 on neurogenesis, we determined that an important component of their programs consists of inhibition of transcription factors [18]. Network analysis showed that transcription regulation and neuronal differentiation are strongly associated, and many transcription factors were identified as main nodes of the identified network. Among the most relevant ones are three transcription factors with known oncogenic roles—MYB, TCF12, and TCF3 (Figure 6B). Other oncogenic factors were identified as targets of miR-124, miR-128, and miR-137 in the genomic analysis (Figure 6C). Agreeing with the proposed idea that associated miRNAs can be more effective in targeting a particular biological process or pathway by expanding the number of affected genes, we observed that the described network contains a combination of targets of individual miRNAs, as well as genes targeted by two or three miRNAs (Figure S3).

To identify further associations between identified target genes and other biological processes affected by the miRNA combination, we conducted an analysis with HumanBase [35]. Three main functional modules were identified. In Module 1, main terms were related to histone modification and DNA repair; Module 2 was strongly linked to angiogenesis; in Module 3, among the main terms were transforming growth factor beta and formation of ribonucleoprotein complex (Figure S4 and Table S6). 

Genes showing increased expression after transfection of the miRNA combination are preferentially associated with protein kinase activity, protein localization and secretion, and cell morphogenesis (Figure S5 and Table S5).

### 2.3. Radio-Sensitization by miRNA Combination

**Figure 7:**
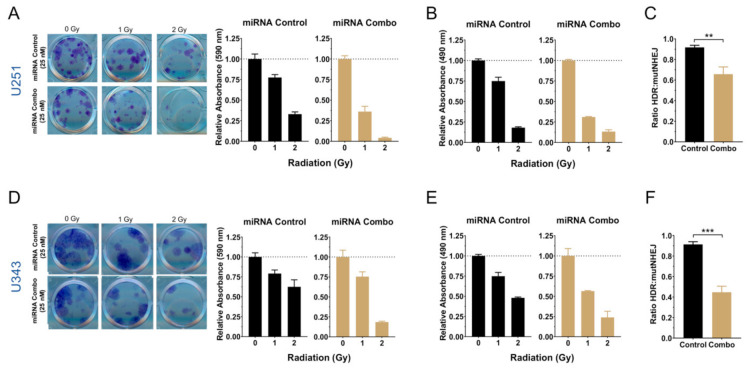
miRNA combo glioblastoma cells. (**A**,**D**) Left: representative aspects of U251 and U343 cells transfected with control or miRNA combination (miR-124, -128, and -137) clonogenic ability following exposure to ionizing radiation; right, quantification of clonogenic assay results. (**B**,**E**) Viability of U251 and U343 cells transfected with control or miRNA combination 48 h after exposure to ionizing radiation. (**C**,**F**) Results of traffic light reporter assays displaying ratios of homologous recombination to mutant NHEJ of U251 and U343 cells transfected with control or miRNA combination 48 h after transfection with the I-SceI plasmid. Student’s *t*-test: ** *p* < 0.01; *** *p* < 0.001.

Radiotherapy is a cornerstone of GBM treatment, along with surgical resection and chemotherapy [27]. Radioresistance is a major cause of recurrence in GBM. GSCs are notoriously radioresistant and sensitizing them to ionizing radiation is critical for therapy. Alterations in DNA damage repair pathways enable GSCs to escape lethal damage [36]. Homologous recombination is particularly important in radioresistance of GSCs, allowing them to repair severe damage caused by therapy [37]. A large number of genes implicated in DNA repair, DNA replication, and cell cycle were altered upon miRNA combination transfection based on the RNA-seq analysis (Tables S5 and S6). This could potentially skew DNA repair pathways that GBM cells normally rely upon and make them more sensitive to treatment with ionizing radiation. We also observed that U251 and U343 cells transfected with 25 nM of the miRNA combination further disrupted the clonogenic ability of these cells and decreased their viability. To better illustrate effects of miRNA combination, we normalized values to the respective nonirradiated controls and present the results for control vs. combination transfected cells side by side (Figure 7A,B,D,E). 

Next, to assess if the miRNA combination altered DNA repair pathways in GBM cells, we used a traffic light reporter assay that indicates the ratio of homologous recombination to mutant prone nonhomologous end joining [38]. The miRNA combination significantly decreased the rate of homologous recombination, suggesting that enhanced radiosensitization by the miRNA combination is due to targeting DNA repair pathways (Figure 7C,F). 

### 2.4. lncRNA Expression Is Affected by miRNA Combination

We identified several downregulated lncRNAs in the overlap of three analyses. We then used our target prediction tool [39] to determine which ones contain putative miRNA binding sites for miR-124, -128, and -137 (Table S3). Among the ones potentially targeted by all three miRNAs, we identified NEAT1, MALAT1, FAM95B1, and AC048341.1 (Figure S6A). NEAT1 and MALAT1 are well characterized lncRNAs and have been implicated in tumor progression, including GBM [40,41]. MALAT1 has been linked to temozolomide resistance in GBM [42]. NEAT1 functions as a sponge for several tumor suppressor miRNAs in glioblastoma [43,44] and influences critical pathways such as Wnt [45]. High NEAT1 expression correlates with larger tumor size, higher WHO grade, prognosis, and recurrence [46]. We have shown that NEAT1 expression increases after GBM cells are exposed to radiation [47]. Although, FAM95B1 is still a poorly characterized lncRNA, one study in papillary thyroid carcinoma showed that FAM95B1 is significantly correlated with cervical lymph node metastasis, tumor staging, and prognosis [48]. FAM95B1 shows higher expression in glioblastoma (TCGA-GBM) compared with normal cortex samples from GTEx and lower-grade glioma (TCGA-LGG). Survival analysis using patient data from TCGA and CCGA available in GlioVis [49] showed that FAM95B1 high expression is linked to poor glioma patient survival (Figure S6B,C).

### 2.5. Establishing Other Examples of miRNA Synergism

**Figure 8:**
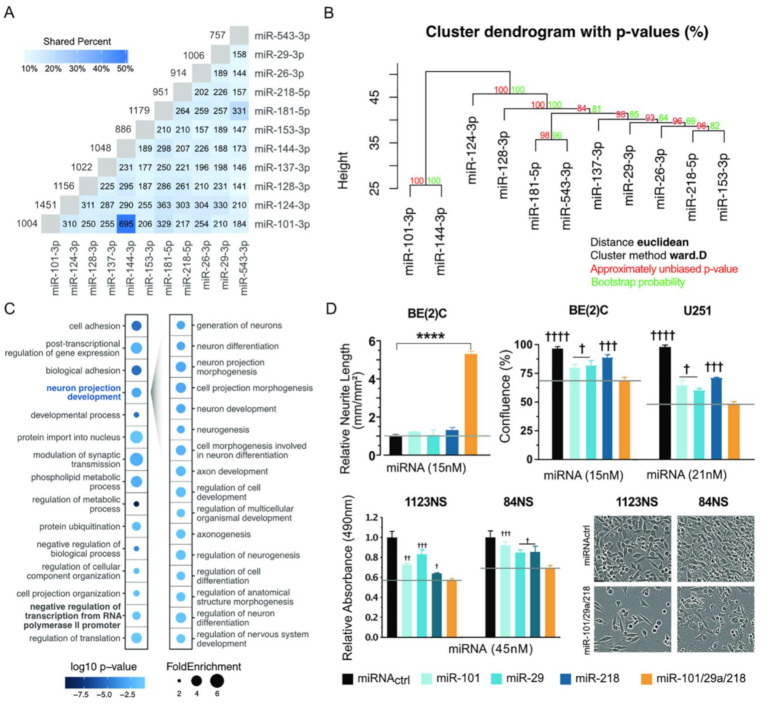
Associated tumor suppressor miRNAs offer other choices of combination treatment. (**A**,**B**) Identified tumor suppressor miRNAs showing strong target overlap according to TargetScan predictions [52]. Numbers indicate overlapping targets and the percentage of overlap is indicated by shading. (**C**) Gene Ontology analysis according to PANTHER [32] shows enriched biological processes for genes predicted to be targeted by at least five of the miRNAs listed in (**A**). (**D**) BE(2)C, U251, 1123NS, and 84NS cells were transfected with the same molecular amount of single miRNA mimics (control, miR-101, miR-29, or miR-218) or combination of three miRNAs. First bar graph: Effect of miRNA transfection (single vs. combination) on BE(2)C cell differentiation at 120 h; neurite outgrowth was used as parameter of neuronal differentiation. Second, third, and fourth bar graphs: Effect of miRNA transfection (single vs. combination) on cell proliferation at 120 h. One-way ANOVA with Tukey test for multiple comparisons was used to analyze results of all experiments. *p*-values for comparisons against miRNA combination: † *p* < 0.05; †† *p <* 0.01; ††† *p* < 0.001; †††† *p* < 0.0001; **** *p* < 0.0001.

Cooperation or synergism between miRNAs has been observed in other systems and scenarios [50]. To identify other miRNA combinations that also could be explored as potential therapies, we looked for examples of tumor suppressor miRNAs sharing a large number of targets. Using all TargetScan predictions [51] for conserved miRNA families, we identified an initial group that included miR-124, miR-128, and miR-137 and shared strong similarity in their target sets. We eliminated from this list miRNAs that do not work as tumor suppressors and included others from nonconserved families that share many targets with the initial group and are relevant to GBM and/or neuroblastoma development. The final list includes 11 miRNAs (Figure 8A,B and Table S7). We performed GO and pathway analyses, focusing on genes predicted to be targeted by at least five miRNAs from the identified group. Similar to the RNA-seq study described above, we found that this group of genes is strongly associated with neuronal differentiation, nervous system development, and transcription regulation (Figure 8C).

To demonstrate that other miRNA combinations could be explored as potential therapies, we selected miR-29a, miR-101, and miR-218 for analysis. These three miRNAs show the same level of target similarity as miR-124, miR-128, and miR-137 and display increased expression during neuronal differentiation. All three have been implicated in GBM and/or neuroblastoma development (Table S7). As before, we determined the impact of individual and combined miRNA transfections on cell proliferation and differentiation, using the same final molecular amount. The miRNA combination produced a stronger impact on proliferation of U251, BE(2)C cells and GSCs 1123NS and 84NS and more effectively promoted differentiation of BE(2)C cells (Figure 8D). Based on both the response additivity and Bliss independence models, the three new miRNAs synergized with CI of 0.731 and 0.443 for U251 and BE(2)C, respectively.

## 3. Discussion

### 3.1. miRNA Combination as a More Robust Approach for Cancer Therapy

Only a few miRNA-based therapies have progressed to clinical trials. Typical issues include the low magnitude of the suppressive effect [53] and the fact that high amounts of miRNA are responsible for a series of off-target effects [54]. Thus, the use of cooperative or synergistic miRNAs can represent a solution, since the combined use of these miRNAs at lower doses could reduce off-target effects, while their synergistic or combinatory effects could provide enough gene-level or network-level suppression to result in a better therapeutic outcome [23,50,55].

We have previously shown that miR-124, miR-128, and miR-137 act synergistically in neurogenesis by targeting a group of common and associated target genes [18]. Moreover, when combined, these three miRNAs produced a much stronger repression of common targets such as SP1 [18]. Cooperation between multiple miRNAs is tied to an array of important cellular processes, such as cell cycle, apoptosis, and differentiation, and their concomitant dysregulation have been linked to disease development [56]. In the setting of cancer, examples of miRNA synergism have been emerging. For instance, miR-34a and miR-15a/16, both tumor suppressors, are often downregulated in the same tumor tissues. In non-small cell lung cancer, miR-34a and miR-15a/16 mimics produced a synergistic effect and induced cell cycle arrest in a Rb-dependent manner [57]. In pediatric acute lymphoblastic leukemia, the combination of miR-125b, miR-100, and miR-99a was more effective in inhibiting target genes associated with chemoresistance than single miRNAs [58]. Similarly, miR-205 and miR-342 increased sensitivity of melanoma and lung cancer cells to a genotoxic cancer drug [59]. On the other hand, miRNA synergism can contribute to tumor development; simultaneous inhibition of the onco-miRs miR-21, miR-23, and miR-27a reduced proliferation of pancreatic ductal adenocarcinoma in vitro and decreased tumor growth more effectively than inhibition of a single miRNAs [60]. In gliomas, simultaneous inhibition of miR-21 and miR-10b sensitized tumor cells in vitro and in vivo to a lower dose of temozolomide [61].

### 3.2. miR-124, miR-128, and miR-137 Target Genes in Differentiation and Development

We and others have previously shown in individual target analyses of miR-124, miR-128, and miR-137 that these three miRNAs activate programs implicated in differentiation while repressing signals that promote stemness [18,20]. Therefore, we expected the miRNA combination to direct GBM and neuroblastoma cells towards differentiation and to impact the viability of cancer stem cells. The RNA-seq analysis identified multiple target genes implicated in neuronal differentiation and nervous system development. Among them were several oncogenic factors, including AKT2, CDH11, TTL, GATA2, NFIB, SUZ12, CDC42, EGFR, and SRC. The last three are the main nodes of the identified network. CDC42 is a small GTPase of the Rho subfamily and has been implicated in cell morphology, cell migration, endocytosis, and cell cycle progression [62]. Knockdown or inhibition of CDC42 in GBM and neuroblastoma cells decreases proliferation and invasion, while increasing chemosensitivity [63,64]. SRC and EGFR are members of the ErbB signaling pathway, which we observed to be enriched in the KEGG pathway analysis. This pathway is activated in numerous tumor types, including GBM and neuroblastoma, with several of its genes often displaying increased expression [65,66]. Targeting ErbB signaling pathway members has been explored as a therapeutic option [67]. KEGG pathway analysis also identified several miRNA target genes regulating pluripotency of stem cells (e.g., MAPK14, DVL2 AKT2, ID3, SMAD5, SMAD9, BMI1, SKIL, TCF3, and PCGF6). Finally, mapping of functional modules identified genes associated with histone and chromatin modification (TAF12, UBE2N, PCGF6, DR1, KDM1A, YEATS4, MYB, PHB, and SUZ12). Epigenetic regulation plays an important role in driving gene signatures implicated in stem cell identity and neuronal differentiation [68].

### 3.3. Combination of miR-124, miR-128, and miR-137 Targets a Transcription Factor Network

miRNAs and transcription factors (TF) concomitantly modulate expression of many target genes and act as central nodes of gene networks, such as those implicated in the origin and progression of neuroblastoma and gliomas [69,70]. Associations between transcription factors and miRNAs are very common in biology and have been described in different systems. For instance, TF-miRNA circuits were well defined and shown to be critical components of *Caenorhabditis elegans* development [71]. Other important examples were characterized in the context of the nervous system [72]. These two kinds of regulatory molecules (miRNAs and TFs) can work together in networks to produce large-scale expression changes in cancer. For instance, miRNA-TF modules have been identified in breast cancer and glioblastoma through computational analyses of genomic datasets [73].

Our previous analysis determined that miR-124, miR-128, and miR-137 regulate numerous transcription factors in the context of neurogenesis [18]. Specificity protein 1 (SP1), which was also identified as a target in the present study, appeared as the central node of the identified transcription factor (TF) network. SP1 is a member of the zinc finger family of transcription factors and participates in numerous cellular processes including chromatin remodeling, cell differentiation and growth, apoptosis, immune response, and DNA damage repair [74]. SP1 is of particular interest due to its many epigenetic and transcription factor partners, such sex hormone receptors, MYCN, p300, histone deacetylases, the BAF complex, and NF-Y [75,76,77,78,79]. Furthermore, SP1 has been shown to function as both a pioneer TF, with the ability to interact with chromatin, and as a TF with enriched binding at transcription-start sites, suggesting an essential role in transcription initiation and as a critical cofactor for TFs [80,81]. SP1 emerges as an important target due to its function and involvement in glioblastoma and neuroblastoma development [82,83], as well as being regulated by all three miRNAs synergistically [18].

Besides SP1, several other oncogenic transcription factors linked to neuronal differentiation, such as MYB, PPARG, TCF3, and TCF12, were identified as miRNA targets downregulated by the combination. MYB or c-MYB belongs to a large family of transcription factors containing HTH DNA-binding domains. MYB regulates the neuroblastoma oncogene MYCN, by controlling its expression and amplification in neuroblastoma lines [84]. In glioblastoma, MYB is an effector of the ZEB-1 pathway, which is implicated in epithelial–mesenchymal transition and key features of cancer stem cells [85]. TCF3 and TCF12 are members of the basic helix-loop-helix (bHLH) E-protein family that recognizes the consensus binding site (E-box) sequence. TCF3 knockdown in GBM cells induced apoptosis and inhibited cell migration via Akt and Erk pathway inhibition [86]. In neuroblastoma, TCF3 shows increased expression in MYCN amplified tumors, and its increased expression is linked to poor prognosis [87]. TCF12 ectopic expression in the hippocampus of young rats led to significant deficits in spatial working memory. Moreover, several Tcf12 rats developed tumors similar to glioblastomas [88]. On the other hand, TCF12 knockdown reduced proliferation and neurosphere formation and altered cell cycle distribution of GBM cells [89].

## 4. Materials and Methods

### 4.1. Glioblastoma and Neuroblastoma Cell Culture and Transfections

Glioblastoma U251 and U343 cells were obtained from the University of Uppsala (Sweden) and cultured in Dulbecco’s modified Eagle’s medium supplemented with 10% fetal bovine serum, 100 U/mL penicillin, and 100 µg/mL streptomycin. Cells were maintained at 37 °C in a 5% CO_2_ atmosphere. Neuroblastoma BE(2)C were obtained from the American Type Culture Collection and Kelly cells were obtained from the Cancer Therapy and Research Center, San Antonio, TX. Neuroblastoma cells were grown in DMEM/F-12 supplemented with 10% fetal bovine serum, 100 U/mL penicillin, and 100 µg/mL streptomycin. Glioma stem cell (GSC) lines (Mesenchymal: 3565, 3128, 1123NS and Proneural: 1919, 19NS, 84NS) were gifts from Drs. Jeremy Rich, Christopher Hubert, and Ichiro Nakano [90,91]. GSCs were cultured in serum-free media consisting of Neurobasal-A media supplemented with B-27, sodium pyruvate, Glutamax, penicillin/streptomycin, 20 ng/mL EGF (ThermoFisher), and 20 ng/mL hFGF (PeproTech). Every 72 h, GSCs were pulsed with EGF/FGF. Dissociation was performed by incubating GSCs with Accutase (ThermoFisher) at room temperature for 10 min. 

miRNA mimics were obtained from Qiagen (Negative Control Mimic: YM00479903, miR-124: YM00471256, miR-128: YM00471226, miR-137: YM00472450, miR-29a-5p: YM00470481, miR-218-5p: YM00471984, miR-101-5p: YM00470928), and were used in the experiments as described below.

### 4.2. Proliferation Assays

Glioblastoma and neuroblastoma cells were reverse-transfected with control or miRNA mimics into 96-well plates at densities ranging from 0.8 to 5 × 10^3^. Growth curves were generated by an automated and noninvasive Incucyte^®^ system live cell imaging system (Essen BioSciences). Cells were imaged every 4–8 h. All experiments were performed with three biological and technical replicates. 

### 4.3. Differentiation Assays

Neuroblastoma cells were reverse-transfected with control or miRNA mimics into 96-well plates at a density of 2.5–5 × 10^3^. Cells were cultured for 120 h and were imaged with the Incucyte^®^ system live cell imaging system. Differentiation was assessed by total neurite length using IncuCyte^®^ NeuroTrack Software Module (Essen BioSciences). All experiments were performed with three biological and technical replicates.

### 4.4. MTS Assays

For cell viability assays, 10^3^ cells per well were plated in a 96-well plate and reverse- transfected with control or miRNA mimics. Then, 96 h after transfection, cell viability was measured using CellTiter-Glo (Promega) following the manufacturer’s instructions. For GSC lines, cells were dissociated and reverse-transfected at a density of 10^4^ cells/well with miRNA control or miRNA mimics and plated into 96-well plates precoated 3 h prior with Geltrex™ LDEV-Free Reduced Growth Factor Basement Membrane Matrix (19.2–28.8 mg/mL). Absorbance was quantified at 490 nm. All experiments were performed with technical triplicates.

### 4.5. Colony Formation Assays

U251 or U343 cells were reverse-transfected with control or miRNA mimics. Then, 24 h later, cells were trypsinized and replated at a density of 0.2 cells/μL. Cells were kept in culture for 10–14 days until colonies were clearly visible. Colonies were fixed with 4% paraformaldehyde solution and visualized by staining with 1% crystal violet. Crystal violet was dissolved from stained plates and absorbance was measured with at 570 nm. All experiments were performed with technical triplicates.

### 4.6. Response to Radiation

U251 or U343 cells were reverse-transfected with control or miRNA mimics. Then, 24 h later, cells were trypsinized and exposed to varying doses of ionizing radiation, using a CP-160 Cabinet X-Radiator (Faxitron X-ray Corp). Cells were then replated, either for MTS assays to assess viability or plated for clonogenic potential. MTS assays were performed as described earlier. Two weeks after irradiation, clonogenic potential was assessed as described earlier. All experiments were performed in triplicate.

### 4.7. Traffic Light Reporter Assays

Traffic Light Reporter (Addgene: 31481) and d20GFP-Donor (Addgene: 31485) plasmids were used to generate lentiviral particles. Lentiviruses were generated and titered as previously described [92]. Traffic light reporter assays were performed as previously described with minor modifications [38]. U343 and U251 cells were first infected with an MOI of 13. Then, 96 h later, cells were replated into 96-well plates at a density of 10^4^. Cells were cotransfected with 100 ng of I-SceI plasmid along with either 50 nM of control or miRNA mimics using Lipofectamine 3000. A total of 72 h after transfection, whole wells were imaged with a Celigo automated cell imager (Nexcelom Bioscience). Total numbers of GFP and RFP positive cells were counted and used to calculate the HDR:mutNHEJ Ratio. The I-SceI plasmid was a kind gift from Dr. Alexander JR Bishop. Experiments were performed in triplicates.

### 4.8. miRNA Correlation and Survival Analysis in Patients

miRNA expression and survival data for the TCGA LGG cohort were obtained from UCSC Xena (http://xena.ucsc.edu) [93]. TCGA GBM data were obtained from (https://gdc.cancer.gov/about-data/publications/gbm_2013) [94]. miRNA and survival data for the neuroblastoma cohort were obtained from the study of Schulte et al. [24]. GBM survival data were obtained from the study by D’Urso et al. (GEO: GSE13030) [25]. Correlation was assessed by Pearson’s correlation coefficient. For survival analysis, miRNA expression was normalized by z-score and the resulting average z-score for each patient was used for log-rank and Kaplan–Meier analysis.

### 4.9. Statistical Analysis—Biological Assays

Statistical significance of differences in proliferation and differentiation at the 120-h endpoint were determined by a one-way ANOVA with a post-hoc Tukey test for multiple comparisons. Synergy combination indices were calculated using (1) linear interaction model CI = (*E*_A_ + *E*_B_ + *E*_C_)/*E*_ABC_; (2) Bliss independence model CI = ((*E*_A_ + *E*_B_ + *E*_C_)-(*E*_A_*E*_B_*E*_C_))/*E*_ABC_. MTS and clonogenic assays were analyzed by one-way ANOVA and a post-hoc Tukey test for multiple comparisons. For radiation experiments, a two-way ANOVA with a post-hoc Tukey test for multiple comparisons was used. Results from the traffic light reporter assays were analyzed with Student’s *t*-tests.

### 4.10. miR-124, miR-128, and miR-137 Target Compilation

miR-124, miR-128, and miR-137 targets were compiled from our previous studies [18]. We also included target genes of the three miRNAs obtained from mirTarBase [95] for which there was strong experimental evidence.

### 4.11. RNA Extraction and RNA Sequencing

BE(2)C, 3565, and 1919 cells were reverse-transfected in technical triplicates with 30 nM of miRNA mimics (control vs. miR-124/miR-128/miR-137 combination). Then, 48 h later, total RNA from transfected cells was extracted using TRIzol reagent (Thermo Fisher) according to the manufacturer’s instructions. RNA was purified and concentrated utilizing RNA Clean and Concentrator-5 kit (Zymo Research). Libraries for RNA sequencing were prepared using TruSeq RNA Library Prep Kit v2 (Illumina), following the manufacturer’s instructions, and sequenced at the GCCRI Genome Sequencing Facility on a HiSeq-3000 sequencer (Illumina). Three biological replicates of control and experimental samples were used in each study.

Data were deposited in European Nucleotide Archive (ENA, www.ebi.ac.uk/ena) with the study identifier PRJEB40058.

### 4.12. miRNA Quantification

Isolated RNA was converted into cDNA utilizing High-Capacity cDNA Reverse Transcription Kit (Thermo Fisher), according to the manufacturer’s instructions. For each miRNA quantified, a specific stem-loop TaqMan primer-probe pair from Thermo Fisher was used: (RNU48: 4427975-001006, miR-124: 4440886-003188_mat, miR-128: 4427975-002216, miR-137: 4427975-001129). Threshold detection cycles (Ct) were presented to display differences in expression between the different cell lines and conditions.

### 4.13. Transcriptomic Analyses

Transcript quantification was performed using Kallisto (v0.43.1, parameters: –bootstrap-samples = 100 –single -l-l 350 -s 10) [96] with insert metrics obtained from the library construction. Gene-level counts were obtained using the R package tximport v1.0.3 [97]. GENCODE v29 (gencodegenes.org) was used as the reference for the human transcriptome. Differential gene expression analyses were carried out with DESeq2 v3.6.2 [98]. Genes were classified as differentially expressed using adjusted *p*-values < 0.05 and |log2FoldChange| ≥ 0.5.

Two main gene lists containing the overlap of the three different studies were generated. The upregulated gene set consists of the genes considered upregulated after transfection of the miRNA combination in at least two studies. The same strategy was used to generate the downregulated gene set. For both sets, only protein coding genes were included and conflicting expression behavior genes (i.e., genes downregulated in one comparison and upregulated in another) were removed from the lists.

### 4.14. Correlations between Binding Sites and Gene Silencing

To assess correlations between silencing and presence or absence of binding sites for each of the three miRNAs, log2FoldChange of downregulated genes and not differentially expressed genes were analyzed accordingly with the number of miRNAs targeting the gene. Wilcoxon tests to analyze differences between the group without miRNA targeting and with at least one target were performed for each cell line.

To evaluate correlations between silencing and the number of binding sites, each gene downregulated in at least two of three studies was assigned with a total amount of miR-124, -138, and -137 binding sites indiscriminately. Binding site data were retrieved from TargetScan [51]. Genes were classified between high and low silencing according to the 1st and 3rd quartile. Genes with Log2FoldChange’s standard deviation greater than 0.5 between studies were removed from the analysis. Wilcoxon tests were used to assess differences between the high silencing and low silencing groups. Data were further categorized as being in a single site or multiple sites and statistical differences between groups were assessed by Chi-squared tests.

### 4.15. Permutation Analyses

To determine if the downregulated sets contain more targets of miR-124, miR-128, and miR-137 than expected by chance, we performed a permutation test with 10,000 iterations. Random samples of the same size as the downregulated sets in all three cell lines (*n* = 253) were extracted from all differentially expressed genes. Next, the percentage of genes targeted by two or more miRNA in the sample was calculated.

### 4.16. LncRNA Analyses

To identify potential interactions between miR-124, miR-128, miR-137, and lncRNAs, a list with the lncRNA downregulated in at least two cell lines was collected. Sequences of lncRNAs were obtained from LNCipedia v5.2 [99]. Binding site prediction for these lncRNAs was performed using miRmate [39]. For further analyses, we created a list of lncRNAs that contained predicted miRNA binding sites for all three miRNAs.

### 4.17. Gene Ontology Analyses

Gene Ontology (GO) enrichment analysis was performed with the PANTHER statistical overrepresentation test webtool [32]. For all analyses, the whole human genome was used as background. For a summarized GO term selection, PANTHER’s term list was inputted to REVIGO [100] and the output table was sorted by uniqueness and dispensability. KEGG pathway enrichment analysis was performed using the ShinyGO web tool [33]. In both analyses, terms and pathways with FDR-adjusted *p*-values < 0.05 were considered enriched.

### 4.18. Transcription Factor and Network Analyses

A list with transcription factors was obtained from Lambert et al. [101] and the network was generated with Cytoscape software [102] using STRING’s protein–protein interaction data [34] (minimum interaction score of 0.7).

### 4.19. Expression of miRNA Targets in Neuroblastoma and Glioblastoma Samples

Expression levels of identified miRNA targets in neuroblastoma samples were investigated. Stage 1 and Stage 4 neuroblastoma expression data were obtained from R2 (r2platform.com). The dataset from Kocak et al. contains microarray data from 266 patients (118 Stage 1 and 148 Stage 4) [103]. Following the procedures available in the R2 differential expression webtool, *p*-values were generated by ANOVA tests between log2 transformed data. Genes were considered differentially expressed if *p*-values (adjusted for false-discovery rates (FDR)) were below 0.01.

Mapped RNA sequencing data from 154 primary glioblastoma (GBM) and 248 primary glioma grade II (LGG) samples were obtained from The Cancer Genome Atlas Program (TCGA). TCGA datasets were first reprocessed using Kallisto [96] with GENCODE v29 (gencodegenes.org) as the reference for the human transcriptome. Gene-level counts were also obtained using Tximport v1.0.3 [97]. Gene-level counts from 465 healthy (frontal) cortex samples were directly obtained from the Genotype-Tissue Expression—GTEx portal v8 (gtexportal.org). Analyses of differential gene expression were performed between glioblastoma and glioma grade II samples, as well as between glioblastoma and healthy cortex samples using the R package DESeq2 [98]. Only genes presenting a |log2FoldChange| ≥ 1 and FDR-adjusted *p*-values < 0.05 were considered differentially expressed.

### 4.20. miRNA In Silico Cluster Identification

Target predictions for all broadly conserved miRNA families were first obtained from TargetScan [52]. We selected miRNA families with at least 1000 predicted targets and generated a list of targets shared by at least two of the selected miRNAs. Finally, the selected miRNA families were hierarchically clustered based on information of their shared targets. The miRNA list was further filtered after a literature search for potential roles in glioblastoma and neuroblastoma. We included additional miRNAs sharing a large number of targets with miRNAs in the cluster. The final dataset contains targets of 11 miRNA (miR-101-3p.2, miR-124-3p.1, miR-128-3p, miR-137, miR-181-5p, miR-218-5p, miR-26-5p, miR-29-3p, miR-144-3p, miR-153, miR-543) collected from TargetScan. Hierarchical clustering was carried out in R by pvclust package v2.2 [104] using Euclidean distance and Ward’s hierarchical clustering method.

## 5. Conclusions

We show here that miR-124, miR-128, and miR-137 can synergize to disrupt cancer-relevant phenotypes and GSC growth and that other combinations of tumor suppressive/proneurogenic miRNAs could also produce a similar effect. An ideal platform to move this concept into the clinic would require a systems approach to identify optimal miRNA combinations and tumor matches based on expression profile and clinical data. However, as exemplified by our study, functional validation and genomic analyses are necessary steps to establish the potential therapeutic use of a miRNA combination. We still know very little regarding mechanisms implicated in miRNA cooperativity or synergism and the functional outcome of these interactions. miRNA cooperation or synergism impacts multiple biological processes including stem cell fate decisions. Several groups have used system biology approaches to integrate multiple layers of genomic information and identify such interactions [23,50,56,105,106].

## Data Availability

The data presented in this study are available in the Supplementary Materials. Sequencing data is available on the European Nucleotide Archive (ENA, www.ebi.ac.uk/ena) with the study identifier PRJEB40058.

## Supplementary Materials

The following are available online at https://www.mdpi.com/2072-6694/13/2/289/s1, Figure S1: miRNA overexpression verification. RT-qPCR of miRNAs following miRNA mimic transfection, Figure S2: miR-124, miR-128 and miR-137 synergize against neuroblastoma cells., Figure S3: The cooperative impact of miR-124, miR-128 and miR-137, Figure S4: Modules of target genes observed in at least two RNA-Seq studies (control vs. miRNA combo) obtained with HumanBase, Figure S5: Gene ontology analysis of upregulated genes upon miRNA combo transfection, Figure S6: Downregulated lncRNAs targeted by miR-124, miR-128 and miR-137, Table S1:List of targets of miR-124, miR-128, and miR-137, Table S2: Results of RNA-Seq analysis (control mimics vs. miR-124, -128, -137 combination) in BE(2)C, GSC 1919, and GSC 3565, Table S3: Overlap of RNA-Seq analyses and identified miRNA target genes, Table S4: Characteristics of miRNA combo identified targets, Table S5: Gene Ontology (GO) and KEGG pathway analyses of all downregulated or upregulated genes identified in at least two RNA-Seq studies (control vs. miRNA combination), Table S6: Regulatory modules of target genes observed in at least two RNA-Seq studies (control vs. miRNA combo) obtained with HumanBase, and Table S7: Associated tumor suppressor miRNAs.
