# Supplementary material for: Synergism of Proneurogenic miRNAs Provides a More Effective Strategy to Target Glioma Stem Cells
Source: Cancers (Basel). 2021 Jan 14;13(2):289. doi: 10.3390/cancers13020289 (PMC7831004; doi:10.3390/cancers13020289)
Supplement: Supplementary file 1 [file cancers-13-00289-s001.zip › cancers-1057215-supplementary.pdf]

cancers

MDPI

Supplementary Materials

Synergism of Proneurogenic miRNAs Provides a More Effective Strategy to Target Glioma Stem Cells

Adam Kosti [1,2], Rodrigo Barreiro [1,3,4], Gabriela D. A. Guardia[3], Shiva Ostadrahimi [1], Erzsebet Kokovay [2], Alexander Pertsemlidis [1,5], Pedro A. F. Galante [3,4] and Luiz O. F. Penalva [1,2,*]

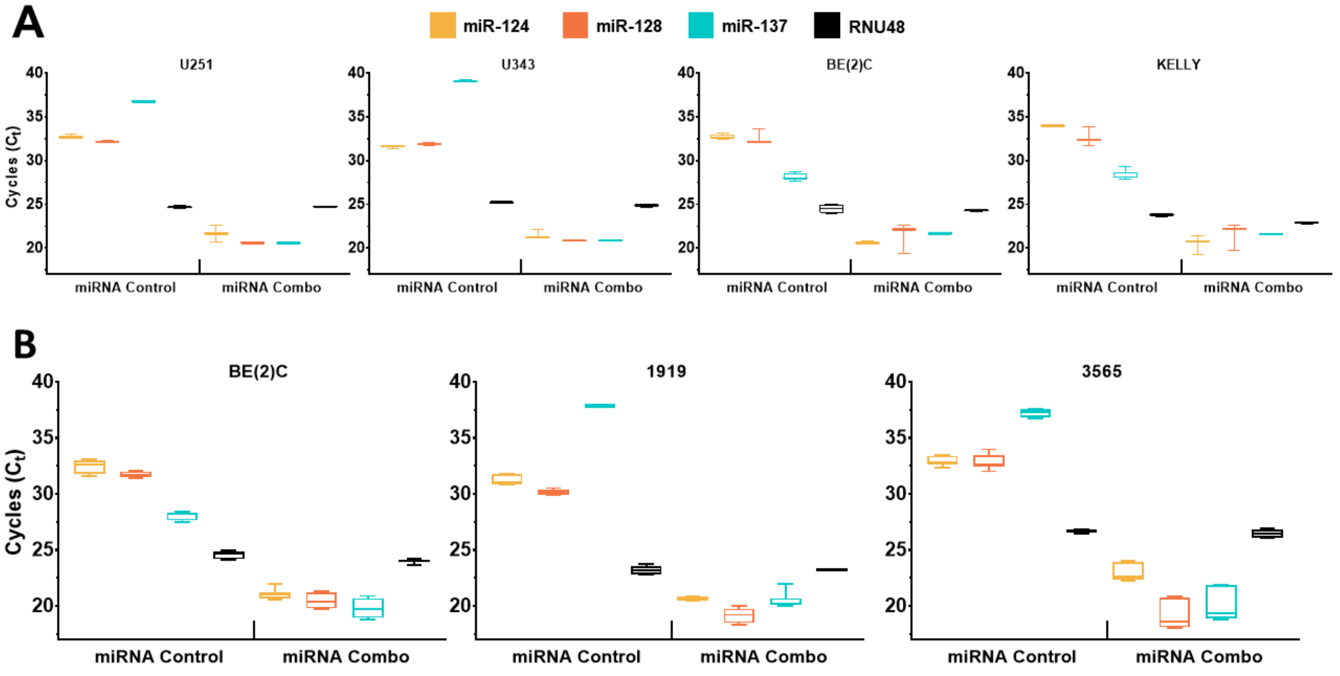

Figure S1. miRNA overexpression verification. RT-qPCR of miRNAs following miRNA mimic transfection. Threshold detection Cycles (Ct) for each small RNA is displayed, with RNU48 serving as an endogenous reference. (**A**) Representative miRNA overexpression following miRNA mimic transfection for *in vitro* experiments featuring cell lines U251, U343, Kelly, and BE(2)C (**Figures 2, 7** and **Figure A2**). (**B**) miRNA overexpression following miRNA mimic transfection for the genomic analyses (**Figures 5, 6, 8**, and **Figures S3-6**).

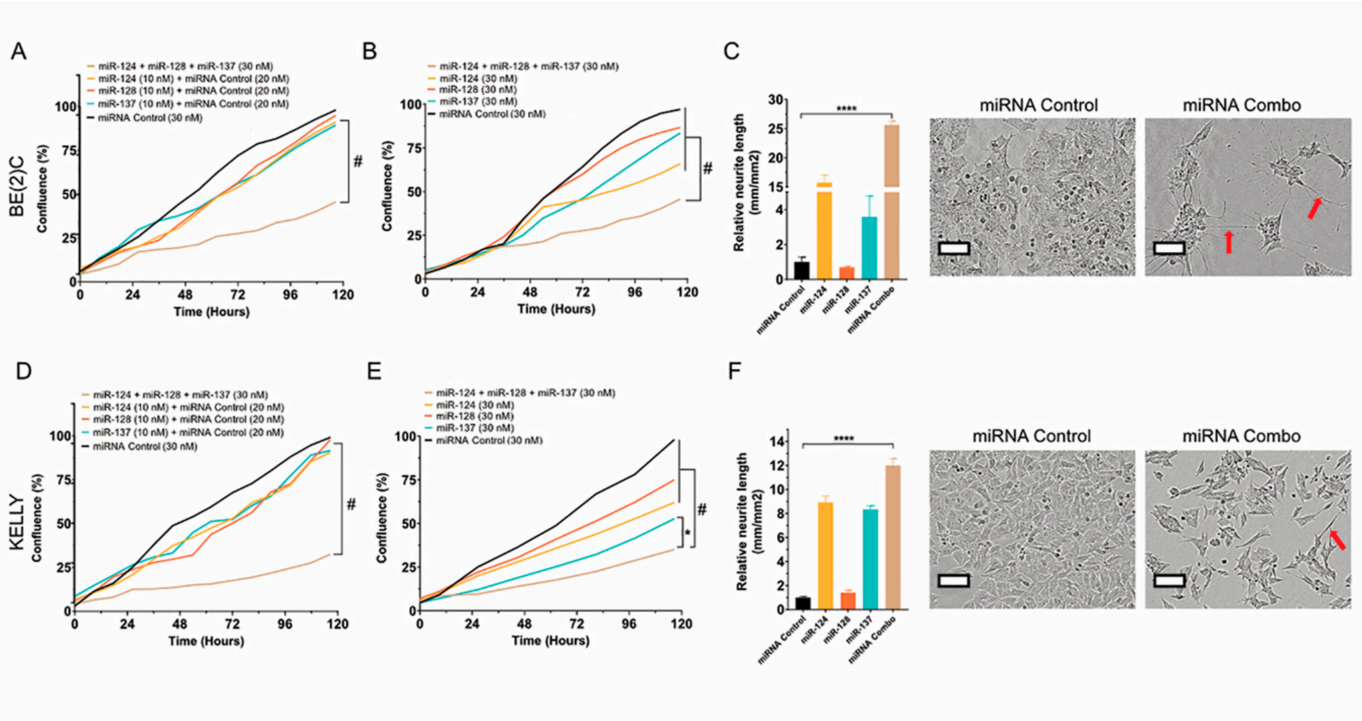

Figure S2. miR-124, miR-128 and miR-137 synergize against neuroblastoma cells. (**A, D**) Cell proliferation with live-cell imaging (Incucyte) over time of neuroblastoma cells following reverse transfection with low concentrations (10 nM) of individual miRNAs and combination of the three miRNAs (total 30 nM). Effect of the combination is greater than expected additivity, indicating synergy as defined by both linear and Bliss synergy models (BE(2)C, Linear CI: 0.355, Bliss CI: 0.354 ; Kelly, Linear CI: 0.264, Bliss CI: 0.263). Tukey test for significance at 120 h, # = $p < 0.0001$. (**B, E**) Effects of miRNA combination on cell proliferation versus individual miRNAs at an equivalent concentration (30 nM). Tukey test for significance at 120 h, * = $p < 0.05$, # = $p < 0.0001$ (**C, F**) Neurite length of neuroblastoma cells 120 hours after reverse transfection with control or miRNA combo mimics (30 nM). A one-way ANOVA with Tukey test for multiple comparisons was utilized, **** = $p < 0.0001$. Representative aspects of neuroblastoma cells with arrows highlighting neurite projections. Scale bar represents 100 μm.

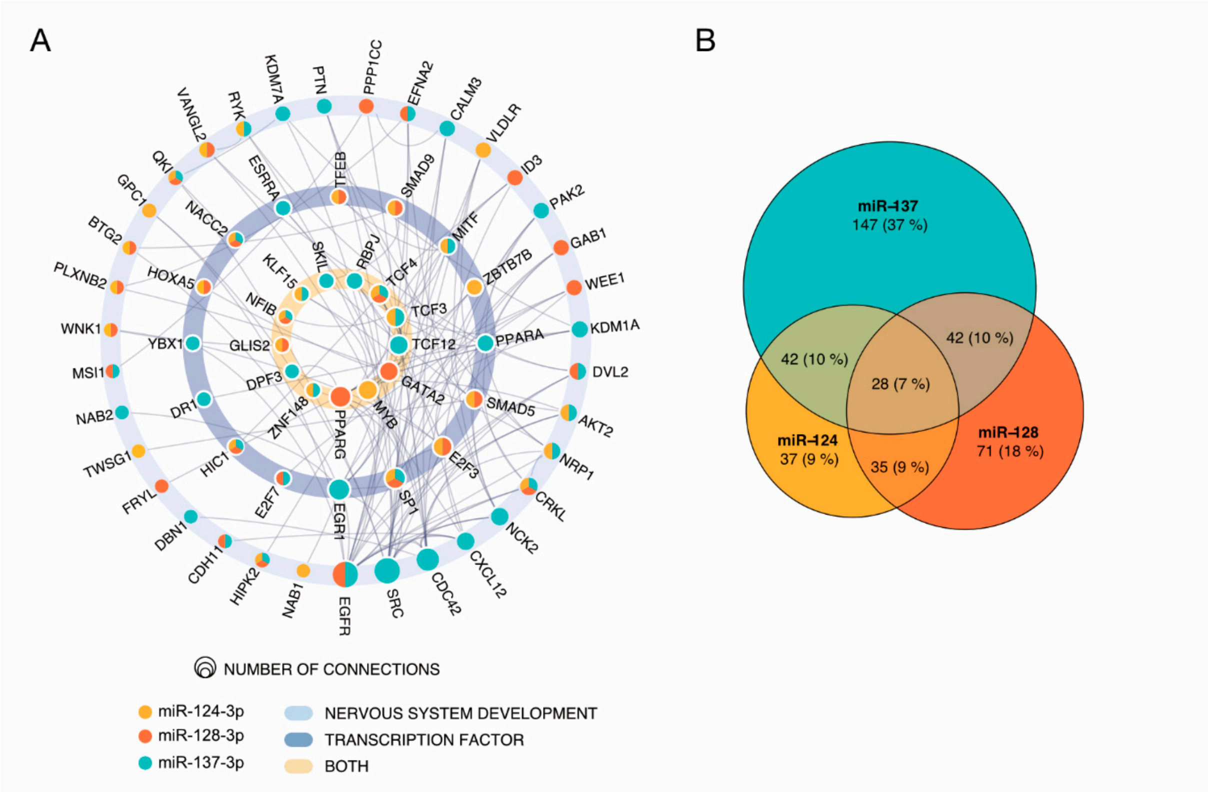

Figure S3. The cooperative impact of miR-124, miR-128 and miR-137. **(A)** Network according to STRING [93] shows targets of miR-124, miR-128 and miR-137 implicated in nervous system development and transcription regulation. miR-124, miR-128, and/or miR-137 targets are labelled with different colors. (**B)** Venn diagram shows number of targets of the three miRNAs identified in at least two studies**.**

TERM_NAME	GO_ID	TERM_Q_VALUE
blood vessel development	GO:0001568	0.013054829
pri-miRNA transcription by RNA polymerase II	GO:0061614	0.013054829
vasculature development	GO:0001944	0.013265393
cardiovascular system development	GO:0072358	0.013265393
angiogenesis	GO:0001525	0.01520793
regulation of angiogenesis	GO:0045765	0.01520793
regulation of receptor biosynthetic process	GO:0010869	0.01520793
blood vessel morphogenesis	GO:0048514	0.016581593
regulation of vasculature development	GO:1901342	0.016581593
receptor biosynthetic process	GO:0032800	0.016581593

TERM_NAME	GO_ID	TERM_Q_VALUE
RNA phosphodiester bond hydrolysis, exonucleolytic	GO:0090503	0.041958816
transforming growth factor beta receptor signaling pathway	GO:0007179	0.049492802
cellular response to biotic stimulus	GO:0071216	0.053288887
epithelial cell differentiation	GO:0030855	0.05357863
fat cell differentiation	GO:0045444	0.063585711
ribonucleoprotein complex assembly	GO:0022618	0.065904703
cellular response to transforming growth factor beta stimulus	GO:0071560	0.067767329
regulation of G2/M transition of mitotic cell cycle	GO:0010389	0.068039864
ribonucleoprotein complex subunit organization	GO:0071826	0.068039864
response to transforming growth factor beta	GO:0071559	0.068058638

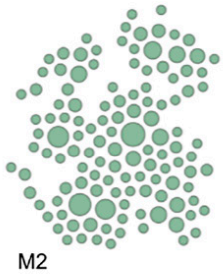

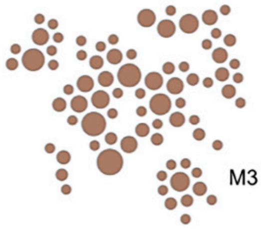

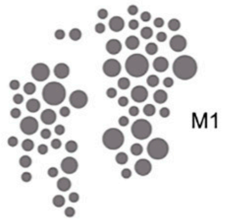

TERM_NAME	GO_ID	TERM_Q_VALUE
histone modification	GO:0016570	0.00821835
covalent chromatin modification	GO:0016569	0.00821835
postreplication repair	GO:0006301	0.00821835
regulation of protein ubiquitination	GO:0031396	0.01520793
regulation of protein modification by small protein conjugation or removal	GO:1903320	0.016581593
regulation of membrane permeability	GO:0090559	0.017201728
regulation of ubiquitin-protein transferase activity	GO:0051438	0.017201728
DNA repair	GO:0006281	0.017710508
histone ubiquitination	GO:0016574	0.017710508
regulation of DNA metabolic process	GO:0051052	0.017710508

Figure S4. Modules of target genes observed in at least two RNA-Seq studies (control vs. miRNA combo) obtained with HumanBase [32]. The 10 top terms in each module are listed.

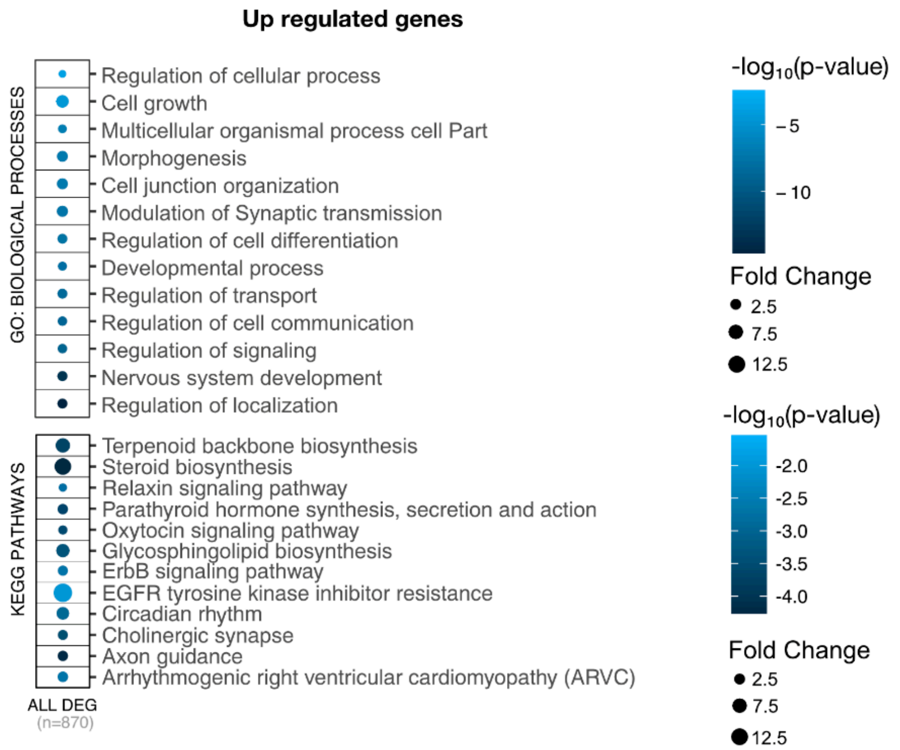

Figure S5. Gene ontology analysis of upregulated genes upon miRNA combo transfection. Enriched biological processes and KEGG pathways identified by Panther and ShinyGO [90] associated with upregulated genes observed in at least two RNA-Seq analyses.

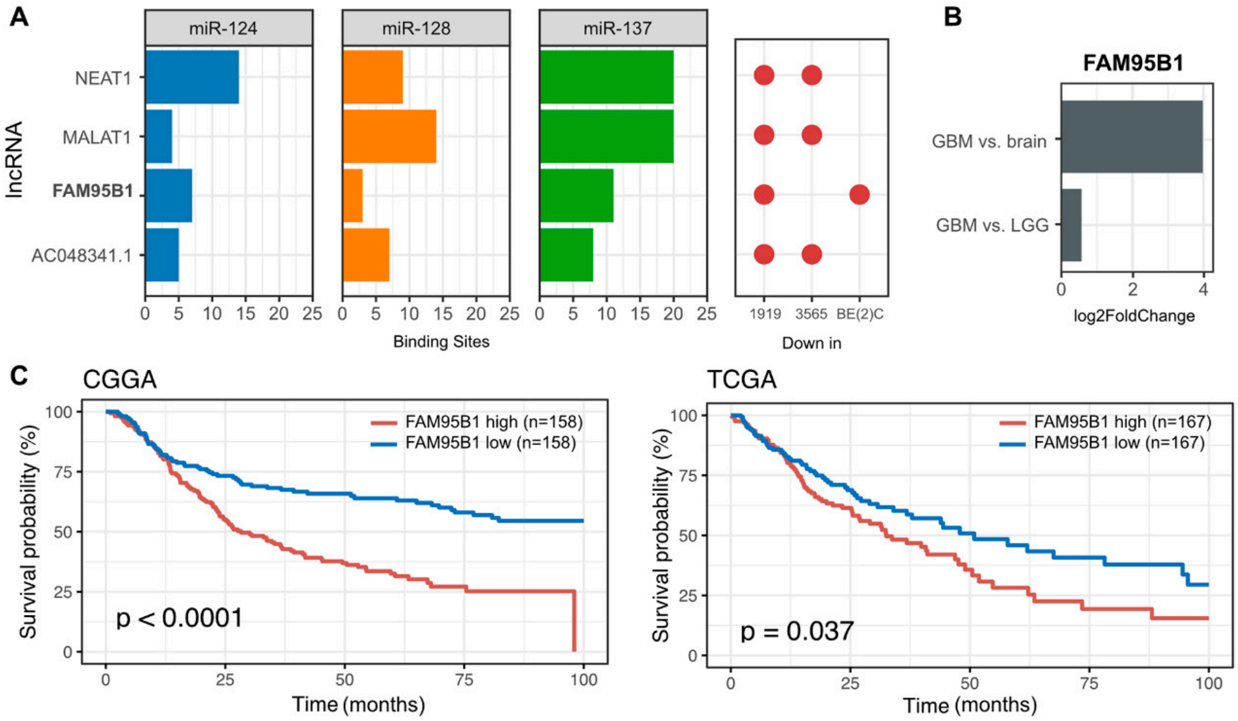

Figure S6. Downregulated lncRNAs targeted by miR-124, miR-128 and miR-137. (**A**) Most relevant lncRNAs downregulated in at least two RNA-Seq studies predicted to be a target of miR-124, miR-128, and miR-137. Bars reflect the number of predicted miRNA sites. (**B**) Expression of FAM95B1 in GBM vs. brain (cortex) and GBM vs. LGG comparisons. (**C**)

Kaplan-Meier curves showing survival rates of patients expressing low vs. high FAM95B1 in samples from the CGGA and TCGA glioma consortia.

Supplementary Tables

Table S1. List of targets of miR-124, miR-128, and miR-137 [18,20,99].

Table S2. Results of RNA-Seq analysis (control mimics vs. miR-124, -128, -137 combination) in BE(2)C, GSC 1919, and GSC 3565.

Table S3. Overlap of RNA-Seq analyses and identified miRNA target genes. Sheets 1-4, genes showing differential expression after miRNA combination transfection in 2 or 3 studies. Sheet 5, downregulated lncRNAs in at least two studies predicted to be a target of miR-124, -128 and/or -137.

Table S4. Characteristics of miRNA combo identified targets. Sheet 1, expression levels of miR-124, -128, and/or -137 targets identified in at least two RNA-Seq studies in GBM vs. normal brain (cortex) and Stage 1 vs. Stage 4 neuroblastoma comparisons. Sheets 2 and 3, Gene Ontology and pathways analyses showing enriched terms associated with miR-124, -128, and/or -137 targets identified in at least two RNA-Seq studies.

Table S5. Gene Ontology (GO) and KEGG pathway analyses of all downregulated or upregulated genes identified in at least two RNA-Seq studies (control vs. miRNA combination).

Table S6. Regulatory modules of target genes observed in at least two RNA-Seq studies (control vs. miRNA combo) obtained with HumanBase [32].

Table S7. Associated tumor suppressor miRNAs. Sheet 1, list of tumor suppressor miRNAs sharing a large number of predicted targets [95]. Sheets 2 and 3, Gene Ontology and KEGG pathway analyses of genes predicted to be targeted by at least 5 miRNAs in Sheet 1. Sheet 4, list of glioblastoma or neuroblastoma-related articles (PMID numbers) for each listed tumor suppressor miRNA.
